# Protein-to-structure pipeline for ambient-temperature *in situ* crystallography at VMXi

**DOI:** 10.1107/S2052252523003810

**Published:** 2023-05-19

**Authors:** Halina Mikolajek, Juan Sanchez-Weatherby, James Sandy, Richard J. Gildea, Ivan Campeotto, Harish Cheruvara, John D. Clarke, Toshana Foster, Sotaro Fujii, Ian T. Paulsen, Bhumika S. Shah, Michael A. Hough

**Affiliations:** a Diamond Light Source Ltd, Harwell Science and Innovation Campus, Didcot OX11 0DE, United Kingdom; b Interdisciplinary Biomedical Research Centre, School of Science and Technology, Nottingham Trent University, Clifton Campus, Nottingham NG11 8NS, United Kingdom; c Bioenergy and Brewery Building, School of Biosciences, University of Nottingham, Sutton Bonington Campus, Nottingham LE12 5RD, United Kingdom; d Membrane Protein Laboratory, Diamond Light Source Ltd, Harwell Science and Innovation Campus, Didcot OX11 0DE, United Kingdom; e Research Complex at Harwell (RCaH), Harwell Science and Innovation Campus, Didcot OX11 0FA, United Kingdom; f The Division of Structural Biology, The Henry Wellcome Building for Genomic Medicine, University of Oxford, Roosevelt Drive, Oxford OX3 7BN, United Kingdom; g Pirbright Institute, Ash Road, Pirbright, Woking GU24 0NF, United Kingdom; h The Rosalind Franklin Institute, Harwell Science and Innovation Campus, Didcot OX11 0QS, United Kingdom; i School of Veterinary Medicine and Science, University of Nottingham, Sutton Bonington Campus, Nottingham LE12 5RD, United Kingdom; j Graduate School of Integrated Sciences for Life, Hiroshima University, Japan; k School of Natural Sciences, Macquarie University, Sydney, Australia; l ARC Centre of Excellence in Synthetic Biology, Macquarie University, Sydney, Australia; Chinese Academy of Sciences, China

**Keywords:** room temperature, *in situ*, multi-crystal, crystallization pipelines, automated data collection, structural biology, radiation damage, X-ray crystallography, VMXi

## Abstract

The utility of X-ray crystal structures determined under ambient-temperature conditions is becoming increasingly recognized. Such experiments can allow protein dynamics to be characterized and are particularly well suited to challenging protein targets that may form fragile crystals that are difficult to cryo-cool. Room-temperature data collection also enables time-resolved experiments. In contrast to the high-throughput highly automated pipelines for determination of structures at cryogenic temperatures widely available at synchrotron beamlines, room-temperature methodology is less mature. Here, the current status of the fully automated ambient-temperature beamline VMXi at Diamond Light Source is described, and a highly efficient pipeline from protein sample to final multi-crystal data analysis and structure determination is shown. The capability of the pipeline is illustrated using a range of user case studies representing different challenges, and from high and lower symmetry space groups and varied crystal sizes. It is also demonstrated that very rapid structure determination from crystals *in situ* within crystallization plates is now routine with minimal user intervention.

## Abstract 2

A pipeline for rapid room-temperature crystal structure determination using a crystallization facility linked to the automated *in situ* beamline VMXi at Diamond Light Source is described.

## 1. Introduction

The importance of determining ambient (room) temperature (RT) and even above RT crystal structures to understand protein function, allostery and the binding of ligands or drugs is becoming ever more recognized (Helliwell, 2020 ▸; Fraser *et al.*, 2011 ▸; Fischer, 2021 ▸). Recent work has shown differences in the response of conformational states to radiation damage between RT and 100 K (Yabukarski *et al.*, 2022 ▸), and in allosteric networks (Keedy *et al.*, 2018 ▸) and ligand binding (Huang *et al.*, 2022 ▸). Significant challenges remain in obtaining such structures without excessive radiation damage or when handling very small or fragile crystals, such as those of membrane proteins, large complexes or viruses. Recent examples of insights from RT crystallography include those for proteins from SARS-CoV2 (*e.g.* Gildea *et al.*, 2022*a* ▸; Kneller *et al.*, 2020*a* ▸,*b* ▸). Increasingly, as seen for example during the COVID-19 pandemic, rapid access to structural data can accelerate biological knowledge and shorten the route to therapeutics, and here the speed of progressing from a purified protein sample to structure determination is critical, allowing for rapid feedback on crystallization conditions or constructs used for protein expression, or for timely iteration in fragment-based drug design. Similarly, rapid assessment of diffraction quality *in situ* allows for the diffracting crystals to be distinguished from those that are easily damaged by manual handling or cryo-cooling. A number of synchrotron facilities have responded to this need by establishing RT data-collection facilities, either for serial crystallography (Horrell *et al.*, 2021 ▸) or *in situ* data collection from crystals within their crystallization plates (Bingel-Erlenmeyer *et al.*, 2011 ▸; Maire *et al.*, 2011 ▸), however the Versatile Macromolecular Crystallography *in situ* (VMXi) beamline at Diamond Light Source was the first dedicated fully automated RT macromolecular crystallography (MX) beamline and also features a ‘pink’ microfocus X-ray beam. The original design and implementation of the beamline have been previously described (Sanchez-Weatherby *et al.*, 2019 ▸) together with examples of initial data measured from thaumatin crystals *in situ* within crystallization plates. Subsequently, other beamlines have been developed with similar features (Okumura *et al.*, 2022 ▸; Martiel *et al.*, 2018 ▸) in parallel with microfluidic and serial crystallographic approaches (Martiel *et al.*, 2019 ▸, 2018 ▸). Here, we describe the current status of the protein crystallization facility (PXF) and VMXi, and provide representative use cases, indicating the benefits of a highly automated RT data-collection pipeline.

The VMXi beamline (Sanchez-Weatherby *et al.*, 2019 ▸) and its associated PXF within the Research Complex at Harwell offer a highly automated pipeline where researchers provide a suitable protein sample that is used for robotic crystallization, monitoring and subsequent *in situ* data collection from crystals in crystallization plates or in thin-film sample delivery systems. The beamline offers a high flux (∼2 × 10^13^ photons s^−1^ at 16 keV) pink microfocus (10 × 10 µm) beam allowing for rapid data collection with up to 60° of plate rotation in a highly automated manner. The VMXi approach can allow for very rapid structure determination as well as assisting in optimizing crystallization conditions, selection of optimal space group for a particular application (*e.g.* ligand soaking), characterization of crystal samples to prepare for X-ray free-electron laser (XFEL) studies, handling different crystal forms within a plate or indeed facilitating drug-binding studies. Efficient and automated data analysis providing rapid feedback is essential to generating high-quality structures efficiently, giving researchers near-to-real-time feedback on their multi-crystal data, for example in assessing when sufficient data have been measured. The beamline recently upgraded its goniometer (see Fig. S1 of the supporting information), which has provided substantially better stability and positional reproducibility, resulting in the ability to routinely collect data on crystals as small as ∼10 µm, either in rotation or serial (*i.e.* stills) data-collection modes.

The beamline concept is that of a fully automated system where *in situ* plates contained within a crystal storage unit with integrated imaging are passed into the beamline hutch for data collection. Crystal positions are pre-selected based on imaging, with a predetermined oscillation range collected from each. Here we present illustrative examples of data measured using VMXi from a variety of proteins comprising both conventional crystallographic standards and ‘real world’ user samples. Data are presented for crystals grown within crystallization plates as well as in LCP medium, and with crystal dimensions ranging from 7 to 150 µm. Alternatively, grid scans may be performed to interrogate a region of interest and assess diffraction quality from crystals that may otherwise be challenging to visually identify.

## 2. Materials and methods

### 2.1. The crystallization laboratory within the Research Complex at Harwell

The PXF is a collaboration between The Research Complex at Harwell, The Rosalind Franklin Institute and Diamond Light Source. The facility has the capability for crystallization of soluble and membrane proteins and currently comprises several robotic crystallization instruments: Mosquito LCP (SPT Labtech) for quick and accurate nanolitre-scale crystallization-drop handling at 4 and 20°C, and Gryphon (Art Robbins Instruments) for crystallization-plate liquid dispensing. The facility also includes an integrated liquid-handling Scorpion robot (Art Robbins Instruments) and Formulator (Formulatrix) to prepare optimization screens, coupled with automated crystallization-plate storage and visualization instruments [Rock Imager and RockMaker (Formulatrix)]. VMXi users have typically undertaken basic crystallization experiments in their home laboratory and thus require a modest quantity (25 µl per plate) of purified protein sample (or bring their crystals already grown in suitable plates), and can then access the laboratory for crystallization within suitable plates for *in situ* data collection. Alternatively, sparse-matrix screening using commercial screens can be used with the above equipment to establish crystallization conditions. For VMXi data collection, Greiner CrystalQuick X (Greiner Bio-One) or MiTeGen *in situ*-1 are currently accepted and are available in the PXF.

### 2.2. Protein preparation and crystallization

#### 2.2.1. Protein crystallization

Beamline benchmark standard proteins were crystallized using established protocols, detailed in the supporting information. The samples from VMXi users had a range of biological origins, and details of their expression and purification are also described in the supporting information. The proteins were crystallized in the PXF in a range of conditions determined individually, using the tools within the PXF and beamline for feedback. Details of the process to determine them are detailed later in the results. Here we describe the final successful crystallization conditions that led to the best structural solution. All experiments used the vapour-diffusion method except for the LCP experiment, where *in meso* crystallization was set up inside the vapour-diffusion chambers of a MiTeGen plate. Phospho­nate ABC type transporter/substrate binding component (PhnD) from *Synechococcus* MITS9220 crystals was grown by mixing 100 nl protein with 100 nl of reservoir [0.1 *M* Tris–HCl pH 8.5, 25%(*w*/*v*) PEG 3350] at a protein concentration of 10 mg ml^−1^. Bovine AbD08 crystals were grown by mixing 100 nl protein (0.15 *M* NaCl, 0.15 *M* Tris–HCl) with 100 nl of reservoir [0.1 *M* Tris (base), 0.1 *M* Bicine at pH 8.5, with precipitants 12%(*v*/*v*) PEG 500 MME, 6%(*w*/*v*) PEG 20000 and additives 0.09 *M* sodium nitrate, 0.09 *M* sodium phosphate dibasic, 0.09 *M* ammonium sulfate] at a concentration of 23 mg ml^−1^ within 96-well CrystalQuick X *in situ* plates at 20°C.

D57-NCOA7 protein sample was screened against multiple crystallization screens (JCSG+, LMB, SG1 and BCS from Molecular Dimensions, and Hampton Index from Hampton Research) at 18°C using 96-well *in situ* plates (MiTeGen) by mixing 50 nl of protein in 0.02 *M* HEPES pH 7.4, 0.1 *M* NaCl and 0.003 *M* TCEP with 100 nl of reservoir solution. The protein crystallized in three days in several conditions: 0.2 *M* di-ammonium hydrogen citrate, 20%(*w*/*v*) PEG 3350 (condition 1); 0.05 *M* zinc acetate dihydrate, 20%(*w*/*v*) PEG 3350 (condition 2); 0.1 *M* citrate pH 5, 20%(*w*/*v*) PEG 6000 (condition 3); 0.2 *M* ammonium sulfate, 0.1 *M* sodium acetate pH 4.6 and 25%(*w*/*v*) PEG 4000 (condition 4); 1.5 *M* lithium sulfate, 0.1 *M* sodium HEPES pH 7.5 (condition 5); 0.2 *M* sodium sulfate, 20%(*w*/*v*) PEG 3350 (condition 6); and 8%(*w*/*v*) PEG 8000, 0.08 *M* potassium phosphate pH 5.6 (condition 7).

Cytochrome *c*′ from *Hydrogeno­philus thermoluteolus* (PHCP) was prepared as described previously (Fujii *et al.*, 2017 ▸). PHCP was dissolved in MilliQ water at a concentration of 10 mg ml^−1^. Equal amounts of protein and a solution of 0.1 *M* sodium acetate pH 4.5, 0.2 *M* lithium sulfate, 30%(*w*/*v*) PEG 8000 were mixed and equilibrated over a well containing 0.1 *M* sodium acetate pH 4.5, 0.2 *M* lithium sulfate, 30%(*w*/*v*) PEG 8000. Cytochrome *c*′ from *Thermus thermophilus* (TTCP) was prepared as described previously (Yoshimi *et al.*, 2022 ▸). TTCP was dissolved in MilliQ water at a concentration of 10 mg ml^−1^. Equal amounts of protein and a solution of 0.1 *M* HEPES pH 7.0, 0.2 *M* NaCl, 20%(*w*/*v*) PEG 6000 were mixed and equilibrated over a well containing 0.1 *M* HEPES pH 7.0, 0.2 *M* NaCl, 20%(*w*/*v*) PEG 6000.

### 2.3. The process within the VMXi beamline, sample selection, data collection and data processing

**Figure 1:**
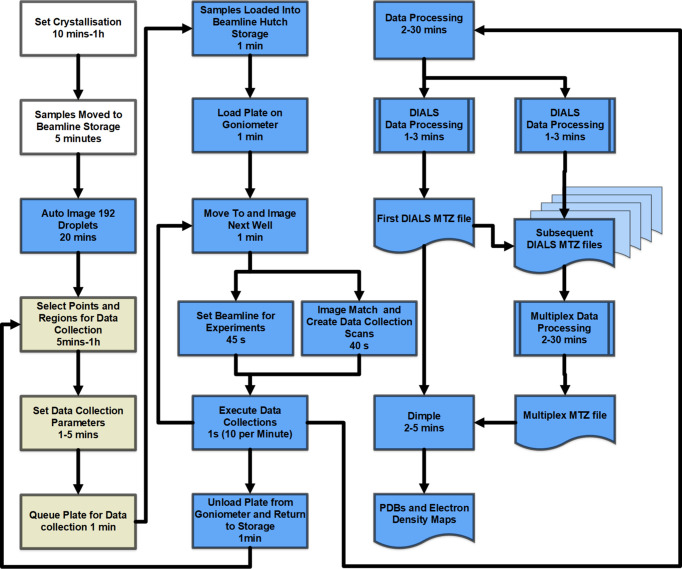
A schematic of the VMXi and PXF workflows. Fully automated steps are shown in blue boxes, required points of user action are shown in white and semi-automated/optional user decision making are shown in tan. Indicative timelines for the different steps are given.

All interactions with the VMXi beamline are conducted via the *SynchWeb* interface (Fisher *et al.*, 2015 ▸) to the information management system *ISPyB* (Delagenière *et al.*, 2011 ▸). Users register samples, access photographic images, mark objects (either as a point of interest or as a region of interest) and set up data collections (as oscillation or raster scans). Following data collection, users can review results and the outputs from automated data-processing pipelines, with the opportunity to reprocess data remotely via this interface. Sample selection, loading and unloading, as well as X-ray data collection and subsequent processing, occur automatically once users have marked samples of interest and queued their plate (see Fig. 1 ▸).

If an area within the crystallization drop (region of interest) is marked then data will be collected by raster scanning the sample in a grid/snake fashion covering the area of interest. Standard parameters for most samples when raster scanning are: 10 µm step size and 0.002 s exposure time using 100% transmitted beam (16 keV, 2 × 10^13^ photons s^−1^ typically), which corresponds to 0.029 MGy, calculated using *RADDOSE*-3D (Zeldin *et al.*, 2013 ▸). Raster scanning is useful to assess the relative diffracting qualities of the objects within the drops *i.e.* resolution and relative intensity using the current automated processing pipelines. Individual diffraction images can be manually analysed for finer assessment of the crystal properties *i.e.* space group, lattice parameters, presence of salts, *etc*. Analysis is being developed that will determine different crystal orientations and crystal composition (*e.g.* whether a drop contains protein, salt, detergent or ‘other’), which will provide more powerful feedback to users on the assessment of their crystallization experiments.

All data presented here were collected using oscillations. Most standard benchmark data were typically collected using predefined parameters of a sweep of 60° of data, at an energy of 12.658 keV, using 0.00178 s exposure time (maximum frame rate of the EIGER2 X 4M), a 0.1° oscillation per frame and 2–5% transmission beam (4 × 10^11^ to 1 × 10^12^ photons s^−1^). These parameters deliver X-ray diffraction weighted doses (DWDs) calculated using *RADDOSE*-3D of the order of 0.73 MGy. Values for specific structures are given in Table 2, and Tables S1 and S2 of the supporting information. This dose is suitable for our standard samples as it delivers suitable data quality and resolution without significantly damaging the samples. Alternatively, and to demonstrate the feasibility of obtaining improved data quality by merging more crystals using lower individual doses, data-collection parameters were modified to include smaller rotation ranges (20° sweeps) and adjusting transmission either to lower (1%) or higher (10%) than the standard. Specific adjusted parameters for the collection of datasets presented here are listed in Tables 1 ▸ and 2 ▸, and are described in the results.

### 2.4. Data processing and analysis

VMXi data processing is triggered automatically upon completion of each measured dataset. Several data-processing pipelines are used to index and merge the diffraction data as used on the other Diamond MX beamlines (Winter *et al.*, 2022 ▸; Winter, 2010 ▸). Successfully processed individual datasets (with *DIALS* via *xia*2) within each crystallization drop trigger a new secondary *xia2.multiplex* pipeline (Gildea *et al.*, 2022*a* ▸) that automatically sorts and merges the individual datasets into a consistent isomorphous dataset suitable for structural solution. Given the data rates and throughput of the beamline, this step is essential in enabling successful data processing and availability of results to users. Here, we demonstrate its potential by applying its use to an example of 39 small wedge thaumatin datasets (Table 2 ▸).

Finally, if a suitable PDB coordinate file or sequence information is provided, the system also carries out several structural solution tasks, from the simple rigid-body refinement via *DIMPLE* (Wojdyr *et al.*, 2013 ▸) (https://ccp4.github.io/dimple/) to full molecular replacement (MR) with either an uploaded PDB file or *AlphaFold* model (Varadi *et al.*, 2022 ▸; Jumper & Hassabis, 2022 ▸) that can be generated automatically for Diamond users from a sequence submitted to *ISPyB* (Gildea *et al.*, 2022*b* ▸). For the results presented in this work, most RT dataset statistics are those taken directly from the automatic pipelines. The only example where manual intervention was utilized was the selection of 39 thaumatin datasets using the reprocessing interface in *ISPyB* but no external software. Details of data processing and structural solution for the final RT and cryo-structures are described in the results and in the supporting information.

## 3. Results

### 3.1. Crystallization and *in situ* data collection

**Figure 2:**
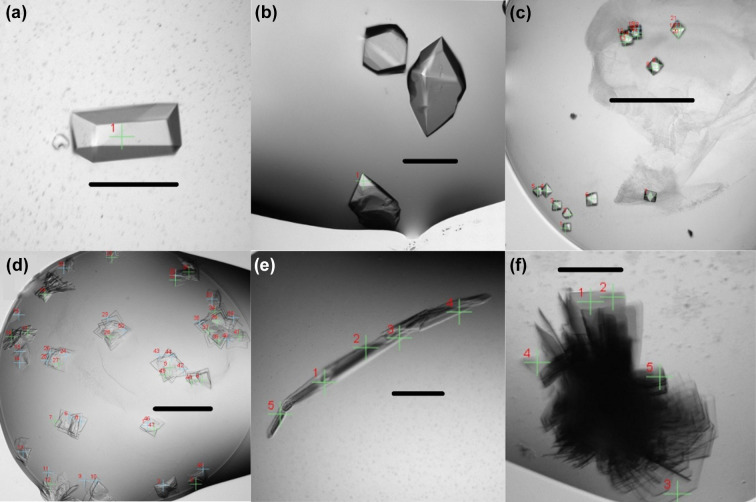
Examples of protein crystals imaged within crystallization plates. Crosshairs represent positions selected for data collection and crystal size is indicated by a scale bar. The crystals are (*a*) PhnD1, (*b*) AbD08, (*c*) NCOA7 crystal form I, (*d*) NCOA7 crystal form II, (*e*) PHCP and (*f*) TTCP. Crystal sizes vary between 10 and 500 µm maximum dimension. The scale bar is ∼100 µm.

Crystallization of the benchmark protein samples were as described previously by using standard crystallization conditions without further optimization and data were subsequently collected as soon as practicable after crystals appeared. These samples offer a range of crystal sizes (7–200 µm) and space groups but are, in general, well diffracting, giving a broad idea of the baseline parameters required to collect good-quality data shown in Table 1 ▸. They are also invaluable to help optimize the automation processes required in the beamline and have helped benchmark the data-processing pipelines built for structural solution. The most challenging test sample in this study, lysozyme in LCP, represented a considerable challenge as, in general, it produces much smaller crystals than the other test cases and is produced in droplets that are much less straightforward to image and align. As a result, working with this sample pushes the envelope of capability of the beamline closer to the more challenging samples we expect the beamline to work with routinely. Examples of crystals for which data are shown in this study are given in Fig. 2 ▸.

Proteins from beamline users were crystallized from scratch using a set of four sparse-matrix commercial screens available at the PXF (JCSG+, SG1, BCS from Molecular Dimensions and Hampton Index from Hampton Research. The assessment of potential hits (typically around 2–8 crystallization hits across the screened plates) was achieved by both visual inspection and directly collecting diffraction data as soon as crystals appeared (hours to days). Depending on their size and visual appearance this either involved raster scanning regions of interest (for microcrystals, clusters, *etc*.) or up to 60° oscillation datasets if crystals were larger and clearly identifiable (above 8 µm in size). In favourable cases of high symmetry, complete data could be obtained from a single 60° dataset, but data quality was improved by merging of multiple datasets. Multi-crystal merging was essential in the majority of cases where a single 60° dataset was incomplete. This allowed for rapid re-optimization and re-screening of crystallization conditions and, in favourable cases and thanks to the automated processing tools, gave full structural data suitable for MR from 4–10 crystals. Suitable crystals were obtained for all targets described here, with illustrative results shown in Fig. 2 ▸. Timelines for moving from provision of purified sample to data collection are indicated in Fig. 1 ▸.

### 3.2. Results for benchmark proteins

For all beamline standard proteins, complete good-quality datasets could typically be collected from 2–10 individual crystals using 60° total rotation for each and 1–5% beam transmission, Tables 1 ▸ and S1, corresponding to a typical dose of 1 MGy. This dose regime typically avoids significant loss of diffraction resolution due to global radiation damage. This is likely to be the case for most future crystal systems used at VMXi, with typical user samples only requiring adjusting transmission and or number of crystals used. Some more dose-sensitive samples may require lower X-ray doses, which can be straightforwardly achieved by simply measuring smaller wedges of *e.g.* 10° and merging data from more crystals.

For certain challenging cases where very low crystal symmetry or plate-like morphology with preferred crystal orientation in the drop may preclude areas of reciprocal space being sampled, we are confident that collection and appropriate merging of a larger number of datasets should help overcome most of these issues. As an example of this capability to merge data from large numbers of crystals, we show in Table S1 how an increased number of crystals (up to 21 for LCP lysozyme and 39 for thaumatin) allowed us to overcome the geometrical resolution limit of the square EIGER 4M 2X detector by adding more high-resolution data from the corners of the detector to effectively increase the resolution limit of the merged dataset. This result highlights the flexible operation of *in situ* data collection at VMXi and its adaptability to the differing requirements of crystal samples, giving confidence that if sufficient good-quality crystals are available most systems will yield suitable data regardless of the space group, geometry and radiation sensitivity in a very simple, fast and streamlined manner. Merged multi-crystal datasets are shown in Table S1 for these proteins, covering a range from low to high symmetry space groups.

### 3.3. Results from typical user protein samples

The streamlined process described above allowed all users to obtain suitable data within a much-shortened time period compared with typical crystallographic workflows involving harvesting and cryo-cooling. Even if samples were also tested under cryo-conditions, the RT *in situ* triaging process helped guide the subsequent plans and ensured focus was targeted to the most promising conditions, saving many hours of harvesting, testing and subsequent optimization. The overall workflow is much simpler and made users much more efficient in their processes. Results are presented here for several case studies from users of the PXF and beamline. Data collection and processing statistics are given in Table 2 ▸. The research aim for these projects was in some cases to test multiple hits from a crystallization screen in order to establish the most promising condition for optimization, and in others to obtain RT structures without the need for crystal handling and to assess differences between RT and 100 K structures (Table S2). The starting point was either crystals within their crystallization plates provided by users or a suitable sample of purified protein from the user that was set up in the PXF.

### 3.4. Lysozyme in lipidic cubic phase (LCP-Lyso)

As an interesting challenge case with samples in LCP and of microcrystal size (8–15 µm), data were collected from lysozyme crystals that had been grown in LCP, Table S1. Despite the challenges for optical imaging within largely opaque LCP medium, 21 crystals were reliably centred within the X-ray beam and an optimized cluster produced from *xia2.multiplex* analysis. Due to the small size of the crystals, high-resolution data to 2.1 Å was achieved by increasing the X-ray transmission to 10% (∼2 × 10^12^ photons s^−1^) but limiting the wedges to only 10° rotation in order to lower the X-ray dose of the merged dataset. The resulting structure obtained using an estimated X-ray dose of 0.48 MGy was highly comparable with previous RT structures obtained from non-LCP crystals and showed no obvious changes in the electron density associated with radiation damage. The disulfide bridges within the structure of lysozyme showed an S—S distance of 2.03 Å, consistent with an intact bond and with no signs of radiation damage in the electron-density maps. Recent studies on radiation damage at RT suggest that fewer site-specific effects are observed in comparison with structures obtained at 100 K with higher doses, and that this may arise because of the decoupling of specific and global radiation damage (Gotthard *et al.*, 2019 ▸).

### 3.5. *Synechococcus* MITS9220 PhnD1 protein

Some VMXi users come with a large number of different protein samples expressed and purified in bulk, and use the beamline to quickly screen and collect data from their crystallization hits. This is a very efficient use of beam time as it helps accelerate projects, and helps iterate between crystallization and data collection very quickly making it easier to find suitable crystallization conditions. As an example, here we show a project that focused on a range of nutrient-uptake proteins isolated from a marine cyano­bacteria (*Synechococcus* MITS9220). This project involved 48 expressed proteins, of which 17 were purified at the Protein Production UK (PPUK) facility (Walter *et al.*, 2005 ▸, 2008 ▸). Once pure protein was available for a number of these proteins, they were put through crystallization screens *en masse* in the PXF, and subsequently potential crystallization hits were identified and analysed by data collection at VMXi. A total of >120 crystallization plates were set, yielding crystal hits for eight proteins, of which one example is described here. As most were novel structures, some crystals from the same crystallization conditions were also cryo-cooled and phased using data from the long-wavelength Diamond beamline I23 (Wagner *et al.*, 2016 ▸). Details of these structures will be published elsewhere.

**Figure 3:**
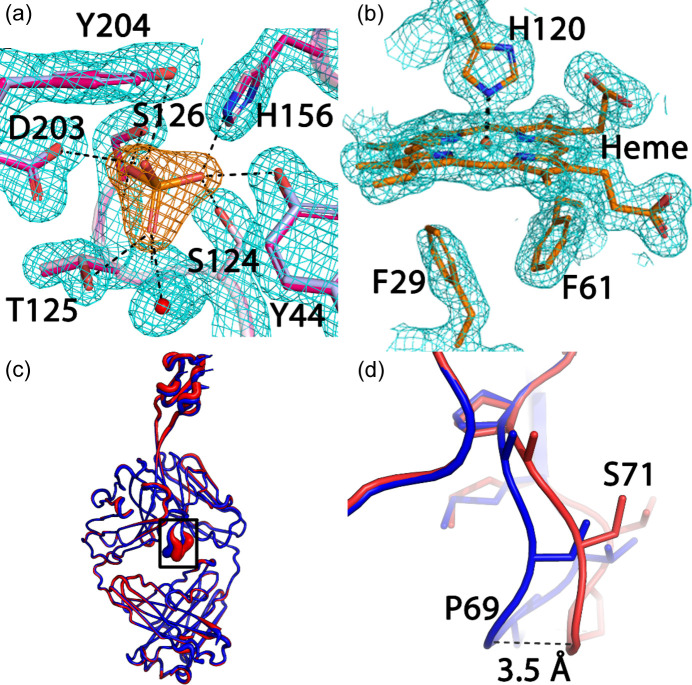
(*a*) An electron-density map (cyan) contoured at 1.8σ for the active site of MITS9220_PhnD1, outlining the interactions between phosphate and MITS9220_PhnD1 residues for the RT structure (magenta, PDB entry 7zck) and the cryo-structure (pink orange, PDB entry 7s6g). The interactions between the side chains and phosphate are shown as black dashed lines. The single buried water is shown as a red sphere. (*b*) An electron-density map (cyan) contoured at 1.3σ for the heme coordination in TTCP RT structure. The heme and surrounding residues are shown as stick models. The coordination bond is shown as a black dashed line. (*c*) A *B*-factor putty representation of the RT (red) and cryo (blue, PDB entry 8bs8) AbD08 structures. The inset view is shown in (*d*), where the largest difference (with a distance of 3.5 Å) was at one of the loops of the VL domain of the AbD08 structures, protruding into space between the four IG domains.

Here, we present the example of *Synechococcus* MITS9220_PhnD1 that yielded a high-quality 1.8 Å resolution RT dataset [see Fig. 2 ▸(*a*) and Table 2 ▸] by merging seven individual crystal datasets, each of 60° rotation. The RT dataset was subsequently phased by MR using the MITS9220_PhnD1 cryo-structure in complex with inorganic phosphate (PDB entry 7s6g; Shah *et al.*, 2023 ▸). As seen for the cryo-structure, the MITS9220_PhnD1 RT crystal structure (PDB entry 7zck; Mikolajek *et al.*, to be published) also has a bound phosphate, resulting in a closed ligand-bound complex [Fig. 3 ▸(*a*)]. The overall fold of the two MITS9220_PhnD1 structures with phosphate is very similar (Fig. S2), with a root mean squared deviation (RMSD) of 0.15 Å. The availability of the two structures in complex with phosphate provided an opportunity to directly compare the ligand binding site and understand if temperature bias potentially led to any differences in the ligand-binding interactions (Fischer, 2021 ▸). Within the crystal structure of MITS9220_PhnD1 RT structure, the four phosphate oxygens are stabilized by ten direct hydrogen bonds with main-chain or side-chain atoms of Tyr44, Ser124, Thr125, Ser126, His156, Asp203 and Tyr204, as well as a water molecule buried deep within the cavity. Given the similarity of the 3D fold of the two MITS9220_PhnD1 structures, unsurprisingly, all four oxygen atoms of the phosphate moiety in the MITS9220_PhnD1 cryo-structure showed an identical hydrogen-bond network [Fig. 3 ▸(*a*)]. Thus, no temperature bias was introduced at the global or local scale for the MITS9220_PhnD1 protein. Even though the average *B* factors for the main/side chain and the solvent are higher for the RT MITS9220_PhnD1 structure compared with the cryo MITS9220_PhnD1 structure, overall, the two structures depict identical dynamic motion and flexibility trends (Fig. S1).

### 3.6. Gas-binding cytochromes *c*′ (PHCP and TTCP)

Other users tend to focus on groups of proteins with homologous functions from a range of microorganisms. This is the case of a group working with cytochromes *c*′. These are two unrelated families of carbon monoxide and nitric oxide binding proteins found in bacteria that contain a *c*-type heme centre (Hough & Andrew, 2015 ▸). Furthermore, they pose a challenge as their heme centre is very sensitive to radiation (Pfanzagl *et al.*, 2020 ▸; Beitlich *et al.*, 2007 ▸; Kekilli *et al.*, 2014 ▸), and thus careful data collection and analysis are crucial. Here we present the results for two different cytochromes *c*′ and their individual challenges.

For the α-helical cytochrome *c*′ protein from *H. thermoluteolus* (PHCP) (Fujii *et al.*, 2017 ▸), a total of two plates were set up yielding one crystal hit for the PHCP protein under the 96 screening conditions. A structure at 1.88 Å resolution was determined from four data wedges, each of 60°, measured at different positions on a single larger crystal (Tables 1 ▸ and 2 ▸). Notably, because of the high-symmetry space group (*P*6_2_22), a single 60° wedge gave a 2.08 Å dataset with 99.4% complete­ness, but adding the three further wedges within *xia2.multiplex* increased completeness to 100% and led to a significant improvement in dataset resolution. In this case, only a single crystal was required for RT structure determination because of the high-symmetry crystal form and the ability to measure data from multiple positions along the crystal. If this had not been the case or radiation damage had been observed, this condition would have been reproduced to produce larger numbers of crystals and data would be sought by merging multiple lower-dose datasets. Phasing was achieved using MR with a previous cryo-structure of PHCP (PDB entry 5b3i) as the search model (Fujii *et al.*, 2017 ▸). The overall RT PHCP structure showed a four-helix bundle, superimposing well with the cryo-structure having an RMSD value of 0.38 Å.

A second cytochrome *c*′ protein from *T. thermophilus* (TTCP) (Yoshimi *et al.*, 2022 ▸), in this case with β-sheet fold, presented a greater challenge. Firstly, crystals grow in clusters of stacked plates [Fig. 2 ▸(*f*)], making it impracticable to harvest and cryo-cool without further optimization of crystallization conditions, but the ability to collect data *in situ* at VMXi made it possible to carefully select positions for data collection around the edges of the cluster [Fig. 2 ▸(*f*)] and the microfocus beam allowed useful datasets to be measured. Despite this, the low-symmetry space group of these crystals (*C*2) required merging four of these carefully selected dataset wedges to yield a 1.75 Å resolution dataset with 93.9% completeness (see Tables 1 ▸ and 2 ▸) without further crystallization optimization or handling. Phasing was achieved using MR with a previous cryo-structure of TTCP as a search model (PDB entry 7ead; Yoshimi *et al.*, 2022 ▸). The electron density revealed a β-sheet fold with a typical five-coordinate heme functional site with two phenyl­alanine residues forming a hydro­phobic cap above the Fe atom (Yoshimi *et al.*, 2022 ▸) [Fig. 3 ▸(*b*)]. A full comparison between the RT and 100 K structures was not feasible because the crystallization conditions as well as space group differed.

### 3.7. Human nuclear receptor coactivator 7

Human Δ57aa-nuclear receptor coactivator 7 (NCOA7) protein (57-148aa) crystallized as a trimer in the asymmetric unit (ASU), unlike the zebrafish homologue, which is a monomer in the ASU (PDB entry 4acj; Blaise *et al.*, 2012 ▸), and the recently published NCOA7 human construct with two trimers in the ASU (PDB entry 7obp; Arnaud-Arnould *et al.*, 2021 ▸). A structure at 2.36 Å resolution was determined from 12 crystals with data wedges, each of 60°, measured at different positions [Figs. 2 ▸(*c*) and 2 ▸(*d*), and Tables 1 ▸ and 2 ▸]. The entire LVPRGS thrombin site was also visible in all three monomers at the N-terminus of Δ57aa-NCOA7 protein, due to the cloning strategy for subcloning into pET15b vector. Crystal contact inspection revealed a different inter-monomer interaction network in Δ57aa-NCOA7 versus the similar construct in PDB entry 7obp, which is likely to be attributed to the different crystallization conditions. Plates were set up yielding crystal hits for the NCOA7 protein under the 96 screening conditions. Comparison between the RT structure and cryo-structures was not feasible due to difference in crystallization condition and space group.

### 3.8. Bovine ultralong antibody (AbD08)

For AbD08, a bovine naïve ultralong antibody (*i.e.* arising from a sorted B cell originating from an animal receiving only a typical course of veterinary vaccines), sparse matrix screening yielded >10 crystallization hits, and the VMXi screening capability was useful to provide fast feedback on diffraction and crystal quality without the user having to go through the lengthy and challenging process of harvesting many crystals. In this case, we were able to rapidly identify promising conditions and collect a complete dataset to 2.2 Å resolution with 96% completeness from four crystals [see Fig. 2 ▸(*b*) and Table 2 ▸]. As done previously, a cryo-structure was also collected (in this case at beamline I04 at Diamond Light Source) that yielded a 1.59 Å dataset (see Table S2). MR was performed for the 100 K data using the protein chain H monomer of bovine ultralong antibody BLV1H12 (PDB entry 4k3d; Wang *et al.*, 2013 ▸) and the protein chain L monomer of bovine antibody B4HC-B13LC (PDB entry 6qn7; Ren *et al.*, 2019 ▸) as search models. MR placed one monomer in the ASU and full structural solution was possible thereafter, despite a 3.5% difference in lattice volume. The final cryo-model (PDB entry 8bs8) was then used as a search model for MR to solve the previously collected RT multi-crystal dataset (see Table S2). Comparison between the two models shows that the RT structure and the cryo-structure align well except in a flexible loop of the Fab VL domain with an RMSD of 3.5 Å [Figs. 3 ▸(*c*) and 3 ▸(*d*)]. In the cryogenic structure, an extensive network of hydrogen bonds involving both protein and water molecules was resolved surrounding this loop (and the greater Fab monomer). The significance of these differences between the two structures is not immediately clear in this case, but may be a consequence of an inherent difference in stability of this loop at both temperatures, thus hinting as a potential link to its biological role. Being able to see and investigate these kinds of differences are some of the advantages that the new capabilities of this VMXi beamline provide and we expect that the information revealed will be invaluable for future projects.

## 4. Discussion

We have described the current status of *in situ* data collection and the associated crystallization pipeline at the VMXi beamline at Diamond Light Source. The pipeline allows routine rapid progress from purified protein to crystal hits, which can be assessed *in situ* in their crystallization plates, together with RT structures obtained from typically a handful of crystals either in mother liquor or LCP. Data collection and/or grid screening from many crystals across a crystallization screen allows for the most promising conditions to be identified based on diffraction rather than visual appearance under a microscope. Importantly, because structures can be determined for many promising hits, desirable space group, crystal packing or other structural properties can guide later optimization. Notably, high-quality complete data are obtainable from crystals as small as 10–20 µm, which would often fall into the category of microcrystals to be studied by serial crystallography methods, even within a more challenging medium such as LCP. Routine RT data collection provides a useful capability that is highly relevant to studying protein dynamics and ligand binding without the complication of cryo-cooling. Tools to fully explore conformational space in RT structures are becoming increasingly available and structures from VMXi promise to greatly increase the number of RT structures available in the PDB.

RT datasets presented here typically used estimated X-ray doses of around 1 MGy, Table 2 ▸. The Garman limit for lifetime of crystals at 100 K is 40 MGy, with RT lifetimes significantly smaller. Global radiation damage assessed by diffracting power data suggests that data collection at VMXi using the standard data-collection parameters is suitable for most crystals, but the capability of *xia2.multiplex* to merge large numbers of datasets straightforwardly means that lower-dose datasets can simply be produced by merging wedges of 10° or smaller with associated doses of <100 kGy.

The very short data-collection times and highly automated nature of the VMXi beamline allow many partial datasets to be measured in a small amount of beam time (often as high as 1500 × 60° wedges from 1–5 plates within an 8 h shift, although variable depending on density of crystals). Often only a small cluster of merged datasets is required to produce a high-quality structure. Merging of data is performed automatically in *xia2.multiplex*, providing quick feedback on data quality and density maps. In principle, this allows for oversampling of structural space, whereby variability in structure may be reflected in different *xia2.multiplex* clusters, or a family of structures may be determined from the whole body of diffraction data allowing for an assessment of the heterogeneity of structural states and the reliability of coordinate positions within each dataset. We have also shown that protein ligand complexes can be effectively and rapidly determined at RT using the VMXi *in situ* pipeline, even for crystals with low symmetry. Effective and automated integration of this with fragment screening has tremendous potential to produce RT structures of protein-fragment complexes, free of any artefacts arising from cryo-cooling. Our recent developments in sample grouping and rapid multi-crystal data processing have now fed into pilot experiments for fragment screening, the results of which will be published in due course. We anticipate RT fragment screening becoming a major activity at VMXi in the coming years.

VMXi has already demonstrated the capability to measure data from crystals of ∼10 µm dimensions, and planned developments for the Diamond-II upgrade will further enhance this ability and enable crystals of substantially smaller size to be used. This will enhance the ability of the instrument to deal with all extremely challenging protein targets where only nano-crystals may be obtained. Data collection from crystals of this size also enables serial crystallography and time-resolved crystallographic approaches at the beamline, which are currently under development.

Coordinate and data files were deposited in the Protein Data Bank with accession codes: 6sel (thermolysin), 6sva (hemoglobin), 6rzp (proteinase K), 6rvo (thaumatin), 8a9d (lysozyme grown in LCP), 7s6g and 7zck (MITS9220_PhnD), 8bs8 and 8cif (AbD08), 8ar9 (NCO), 8brl (PHCP), and 8brk (TTCP).

## 5. Related literature

The following references are cited in the supporting information for this article: Adams *et al.* (2011) ▸, Aherne *et al.* (2012) ▸, Caffrey & Cherezov (2009) ▸, Cheng *et al.* (1998) ▸, Emsley & Cowtan (2004) ▸, Evans & Murshudov (2013) ▸, Kabsch (2010) ▸, Ma *et al.* (2016) ▸, McCoy *et al.* (2007) ▸, Murshudov *et al.* (2011) ▸, Nettleship *et al.* (2009) ▸, Perrakis *et al.* (2001) ▸, Sambongi *et al.* (1996) ▸, Studier (2005) ▸, Williams *et al.* (2018) ▸, Xue *et al.* (2010) ▸, Yuan *et al.* (2016) ▸.

## Supplementary Material

Supporting information. DOI: 10.1107/S2052252523003810/lz5063sup1.pdf

PDB reference: PHCP at room temperature, 8brl

PDB reference: thaumatin at room temperature, 6rvo

PDB reference: haemoglobin at room temperature, 6sva

PDB reference: ABD08 at room temperature, 8cif

PDB reference: TTCP at room temperature, 8brk

PDB reference: thermolysin at room temperature, 6sel

PDB reference: PHND at 100 K, 7sg6

PDB reference: ABD08 at 100 K, 8bs8

PDB reference: proteinase K at room temperature, 6rzp

PDB reference: lysozyme LCP at room temperature, 8a9d

PDB reference: PHND at room temperature, 7zck

PDB reference: NCO at room temperature, 8ar9

## Figures and Tables

**Table 1:** Summary of user and standard datasets measured at VMXi

Sample	Number of crystals merged	Resolution (Å)	PDB entry	
Proteinase K	4	2.20	6rzp	
Thaumatin	39	1.97	6rvo	
Thermolysin	3	2.20	6sel	
Haemoglobin	7	1.92	6sva	
Lysozyme (LCP)	21	2.10	8a9d	
PhnD	7	1.80	7zck	
AbD08	4	2.20	8cif	
NCO	12	2.36	8ar9	
PHCP	4	1.88	8brl	
TTCP	4	1.75	8brk	

**Table 2:** Summary of example user VMXi RT datasets Values in parentheses represent the high-resolution shell.

	MITS9220_PhnD1	AbD08	NCOA7	PHCP	TTCP
Number of crystals	7	4	12	4	4
DWD (MGy)	0.61	0.53	1.01	1.05	1.37
Resolution range (Å)	53.17–1.80 (1.83–1.80)	58.25–2.20 (2.26–2.20)	46.38–2.36 (2.4–2.36)	56.03–1.88 (1.91–1.88)	35.12–1.75 (1.78–1.75)
Space group	*C*2	*P*2_1_2_1_2_1_	*C*2	*P*6_2_22	*C*2
Unit-cell parameters					
*a*, *b*, *c* (Å)	65, 40.7, 106.5	64.2, 71.6, 100.2	100.0, 54.8 44.3	83.5, 83.5, 88.6	81.5, 39.0, 41.5
α, β, γ (°)	90, 92.7, 90	90, 90, 90	90, 119.5, 90	90, 90, 120	90, 97.2, 90
Unique reflections	26032 (1271)	23145 (959)	8049 (420)	15440 (750)	12429 (490)
Multiplicity	8.7 (5.5)	5.1 (4.1)	13.9 (13.3)	22.3 (15.4)	4.4 (2.8)
Completeness (%)	99.8 (97.0)	96.1 (95.8)	92.55 (94.1)	100.0 (100.0)	93.9 (74.7)
〈*I*/σ(*I*)〉	14.4 (1.0)	12.5 (2.3)	5.7 (1.3)	7.9 (0.6)	9.9 (0.9)
Wilson *B* factor (Å ^2^)	24.58	31.53	30.8	19.50	15.38
*R*_merge_	0.108 (1.215)	0.297 (1.851)	0.483 (2.335)	0.459 (5.496)	0.151 (1.171)
*R*_p.i.m._	0.055 (0.782)	0.13 (0.965)	0.13 (0.654)	0.093 (1.401)	0.076 (0.740)
CC_1/2_	0.998 (0.416)	0.985 (0.225)	0.949 (0.261)	0.995 (0.258)	0.990 (0.364)
Reflections used in refinement	24584 (1650)	23012 (1127)	7958 (775)	15397 (777)	12425 (614)
*R*_work_	0.152	0.177	0.196	0.180	0.163
*R*_free_	0.196	0.228	0.259	0.203	0.197
Protein residues	273	460	163	135	133
RMS bonds (Å)	0.0117	0.0091	0.0090	0.0133	0.0118
RMS angles (°)	1.62	1.52	1.61	2.31	2.22
Ramachandran favoured (%)	98.9	96.3	95.7	99.2	100.0
Average *B* factor (Å^2^)	44.2	40.1	26.8	33.0	25.0
