# Supplementary material for: Protein-to-structure pipeline for ambient-temperature in situ crystallography at VMXi
Source: IUCrJ. 2023 May 19;10(Pt 4):420–9. doi: 10.1107/S2052252523003810 (PMC10324489; doi:10.1107/S2052252523003810)
Supplement: Supplementary file 1 [file m-10-00420-sup1.pdf]

IUCrJ

Volume 10 (2023)

Supporting information for article:

Protein-to-structure pipeline for ambient-temperature *in situ* crystallography at VMXi

Halina Mikolajek, Juan Sanchez-Weatherby, James Sandy, Richard J. Gildea, Ivan Campeotto, Harish Cheruvara, John D. Clarke, Toshana Foster, Sotaro Fujii, Ian T. Paulsen, Bhumika S. Shah and Michael A. Hough

S1. SUPPLEMENTARY ONLINE DATA

Table S1 Example datasets from beamline standard protein crystals. All datasets were measured at 0.9795 Å X-ray wavelength, apart from the LCP Lysozyme which was collected at 0.7749 Å X-ray wavelength. Values in parentheses represent the high-resolution shell.

	Proteinase K	Thaumatin	Thermolysin	Haemoglobin	LCP Lysozyme
**Number of crystals**	4	39	3	7	21
**DWD Dose (MGy)**	0.89	0.84	0.89	1.15	0.48
**Resolution range (Å)**	57.13-2.20 (2.28- 2.20)	58.56–1.97 (2.0-1.97)	81.02–2.20 (2.26–2.20)	50.64–1.92 (2.02– 1.92)	55.50–2.10 (2.26–2.10)
**Space group**	$P4_32_12$	$P4_12_12$	$P6_122$	C2	$P4_12_12$
**Unit cell (Å)**	68.4, 68.4, 104.0	58.6, 58.6, 151.5	93.6, 93.6, 130.6	108.5, 63.0, 54.6	78.5, 78.5, 38.1
α, β, γ (°)	90, 90, 90	90, 90, 90	90, 90, 120	90, 111.1, 90	90, 90, 90
**Unique reflections**	13162 (631)	19537 (950)	17829 (875)	21606 (129)	7390 (371)
**Multiplicity**	14.8 (7.1)	39.1 (8.6)	14 (6.9)	6.1 (1.0)	15.2 (16.0)
**Completeness (%)**	100.0 (100.0)	100.0 (99.8)	100.0 (100.0)	81.8 (10.6)	100.0 (100.0)
**Mean I/sigma(I)**	30.5 (12.2)	8.6 (0.8)	14 (3.1)	9.3 (1.3)	6.1 (1.2)
**Wilson B-factor (Å²)**	10.0	24.9	22.6	24.7	24.4
**R-merge**	0.064 (0.132)	0.417 (2.392)	0.136 (0.575)	0.114 (0.383)	0.524 (3.260)
**R-pim**	0.017 (0.052)	0.062 (0.780)	0.074 (0.613)	0.038 (0.631)	0.137 (0.834)
**$CC_{1/2}$**	0.99 (0.98)	0.99 (0.24)	0.99 (0.87)	0.99 (0.60)	1.0 (0.30)
**Reflections used in refinement**	13109 (1287)	19444 (1683)	16003 (1557)	20573 (284)	7349 (642)
**R-work**	0.11 (0.090)	0.1417 (0.294)	0.1291 (0.161)	0.1408 (0.221)	0.1935 (0.285)
**R-free**	0.161 (0.156)	0.1762 (0.315)	0.169 (0.200)	0.1733 (0.265)	0.249 (0.371)
**Protein residues**	279	206	316	285	129
**RMS bonds (Å)**	0.009	0.010	0.008	0.018	0.002

**RMS angles (°)**	0.895	0.866	0.961	1.431	0.361
**Ramachandran favored (%)**	97.11	98.04	97.00	96.70	96.85
**Average B-factor (Å^2)**	14.2	27.4	28.0	39.0	31.5
**PDB code**	6RZP	6RVO	6SEL	6SVA	8A9D
**Starting File For MR**	2ID8	1RQW	5A3Y	5C6E	2LYZ

Table S2 Comparison of RT and 100K data for selected samples. Values in parentheses represent the high-resolution shell.

	**PhnD RT**	**PhnD Cryo**	**AbD08 RT**	**AbD08 Cryo**
**Number of crystals**	7	1	4	1
**Resolution range** (Å)	53.17-1.8 (1.83-1.8)	106.61-2.02 (2.07-2.02)	58.25-2.2 (2.26-2.2)	57.64-1.59 (1.62-1.59)
**Space group**	C2	C2	P212121	P212121
**Unit cell (Å)**	65.0, 40.7, 106.5	63.6, 40.7, 106.7	64.2, 71.5, 100.2	63.5, 71.1, 98.4
α, β, γ (°)	90, 92.7, 90	90, 92.1, 90	90, 90, 90	90, 90, 90
**Unique reflections**	26032 (1271)	18101 (1281)	23145 (959)	60610 (4385)
**Multiplicity**	8.7 (5.5)	21.2 (7.7)	5.1 (4.1)	6.7 (6.8)
**Completeness (%)**	99.8 (97.0)	99.7 (95.6)	96.1 (95.8)	99.9 (99.9)
**Mean I/sigma(I)**	14.4 (1.0)	40.5 (12.2)	12.5 (2.3)	14 (1.3)
**Wilson B-factor (Å^2)**	24.58	20.06	31.50	27.26
**R-merge**	0.108 (1.215)	0.061 (0.113)	0.297 (1.851)	0.062 (1.144)
**R-pim**	0.055 (0.782)	0.012 (0.042)	0.13 (0.965)	0.024 (0.526)
**$CC_{1/2}$**	0.998 (0.416)	1.0 (0.994)	0.985 (0.225)	1.0 (0.600)
**R-work**	0.152	0.155	0.177	0.176
**R-free**	0.195	0.196	0.228	0.220
**Protein residues**	273	273	460	474
**RMS bonds (Å)**	0.0117	0.0145	0.0091	0.0128
**RMS angles (°)**	1.620	1.850	1.522	1.774
**Ramachandran favored (%)**	98.90	98.17	96.30	96.83
**Average B-factor (Å^2)**	44.20	33.47	40.12	23.26
**Coordinate File**	7ZCK	7S6G	8CIF	8BS8
**Starting File For MR**	7S6G	Direct method	8BS8	4K3D chainH, 6QN7 chainL

S2. SUPPLEMENTARY METHODS

S2.1. Protein Production, Crystallisation and comparative 100K data collection

Beamline benchmark standard proteins were purchased from Calbiochem (Thermolysin) or Sigma Aldrich (all other proteins). All standard crystallisation experiments were set up using a Mosquito LCP (SPT Labtech) liquid handling robot using 100 nl protein + 100 nl mother liquor in each drop volume and 30 µl of precipitant solution in each well. The sitting drop vapour diffusion method was used and all trays were stored at 293 K and imaged in a RI1000 Imager System (Formulatrix) located in the VMXi beamline prior data collection. All crystallisation experiments were setup in either CrystalQuick™X Plate (Greiner) (Bingel-Erlenmeyer *et al.*, 2011, le Maire *et al.*, 2011) or In-Situ-1 (MiTeGen) as they offer lower X-ray background and are compatible with the VMXi automation pipeline. Thermolysin (THERM) from *Bacillus thermoproteolyticus* was dissolved in 0.05 M MES pH 6.0, 45% (v/v) DMSO to a concentration of 100 mg/ml. Equal amounts of the protein solution and a solution containing 0.05 M MES pH 6.0, 0.05 M sodium chloride, 45% (v/v) Dimethyl Sulfoxide (DMSO) were mixed and equilibrated over a well containing 1.2 M ammonium sulphate. Thaumatin (THAUM) from *Thaumatococcus daniellii* at a concentration of 40 mg/ml was dissolved in a solution of saturated dithionitrobenzoate (DTNB). Equal amounts of protein and a solution of 0.1 M sodium citrate pH 5.6, 0.75 M sodium/potassium tartrate were mixed and equilibrated over a well containing 0.1 M sodium citrate pH 5.6, 0.75 M sodium/potassium tartrate.

Proteinase K (PK) from *Parengyodontium album* was dissolved in 0.025 M sodium HEPES pH 7.5 at a concentration of 20 mg/ml. Equal amounts of protein and a solution of 1.2 M ammonium sulphate, 0.1 M sodium cacodylate pH 6.2, 25% glycerol were mixed and equilibrated over a well containing 1.2 M ammonium sulphate, 0.1 M sodium cacodylate pH 6.2, 25% glycerol. Haemoglobin (Hb) from *Equus caballus* was dissolved in ddH_2O at a concentration of 20 mg/ml. Equal amounts of protein and a solution of either 0.2 M sodium sulphate, 20% (w/v) PEG 3350, 0.1 M Bis-Tris Propane or 0.2 M sodium sulphate, 20% (w/v) PEG 3350 were mixed and equilibrated over a well containing 0.2 M sodium sulphate, 20% (w/v) PEG 3350, 0.1 M Bis-Tris Propane or 0.2 M sodium sulphate, 20% (w/v) PEG 3350.

LCP Lysozyme (Lyso) crystallisation is slightly different to the other standard proteins and resembles those typically used for membrane proteins. The Lipidic Cubic Phase (LCP) was prepared by combining the monoolein (MAG 9.9) lipid with the lysozyme in a ratio of 3:2 (w/v) (Cheng *et al.*, 1998, Caffrey & Cherezov, 2009, Aherne *et al.*, 2012). Lysozyme at a concentration of 50 mg/ml was used and the protein solution in PBS was centrifuged at 20000 x g at 4 °C for 30 mins prior to the set up. The monoolein was melted in a heat block at 38 °C to facilitate loading into a syringe. Ferrules of the correct size fitted firmly into two glass gas tight Hamilton syringes of different sizes (100 µl for lipid and 50 µl for protein). The protein and monoolein were mixed in the previously mentioned ratio in Hamilton syringes until the mixture became optically clear and nonbirefringent (~5 mins). The syringe with LCP

was attached to the Mosquito LCP robot equipped with a humidity chamber (SPT Labtech) and dispensed onto MiTeGen plate using 1:16 (50 nl LCP to 800 nl precipitant) ratio. The precipitant solution consisted of 1 M sodium chloride, 0.1 M sodium acetate buffer pH 4.5 and 30% (v/v) PEG 400 (Aherne *et al.*, 2012). The crystals appeared within an hour. Crystals sized 8 – 15 microns were marked for data collection using the SynchWeb interface and 10° of data were collected from each crystal using a transmission of 10%. Out of 36 crystals marked, 21 were scaled and integrated using DIALS and were then combined automatically by the xia2.multiplex pipeline. This resulted in a dataset with 100% completeness to 2.1 Å. Molecular replacement was carried out in PHASER (McCoy *et al.*, 2007), using the deposited lysozyme structure 2LYZ (Diamond, R., 1974) as a search model. Phenix.refine was used in an iterative fashion in combination with COOT to refine the structure and place water molecules, leading to a final R_{work} and R_{free} of 19.35% and 24.90%. Coordinates were validated using the wwPDB validation service (https://validate-rcsb-2.wwpdb.org) and deposited in the PDB (8A9D).

S2.2. *Synechococcus* MITS9220 PhnD1

Synechococcus MITS9220_PhnD1 was expressed to high density using the autoinduction method (Studier, 2005) in 1 L culture medium in an *E. coli* Lemo™ strain (BL21 derivative, NE Biolabs). Cells were harvested (4,000 x *g*, 45 minutes), resuspended in the PhnD1 buffer (0.05 M Tris pH 7.5, 0.03 M Imidazole, 0.5 M NaCl) and 0.2% Tween, and lysed using a cell disruptor (Constant Systems) at 30 Kpsi. Clarified cell lysates (following 40 min centrifugation at 11,000 × *g* and 0.2 µm filtration) were loaded onto a prepacked 1 mL Ni-NTA column (GE Healthcare) pre-equilibrated in the same buffer. The column was then washed with 100 column volumes of PhnD1 buffer, and the protein was eluted in the PhnD1 buffer and 0.5 M imidazole. Eluate fractions were pooled and further purified using SEC (Superdex HiLoad 200 16/600 column, GE Healthcare) in 0.02 M Tris pH 7.5, 0.2 M NaCl supplemented with reducing agent tris(2-carboxyethyl) phosphine (TCEP, 0.001 M). Protein-containing fractions were pooled for proteolytic cleavage of the His_6-tag using a 3C protease enzyme. The cleaved protein fraction was recovered using a reverse IMAC step, collecting the flowthrough. The purity of the cleaved protein was verified by SDS-PAGE, showing a single band at ~30 kDa. The protein was concentrated to 10 mg/mL using centrifugation (Centricon 10 kDa molecular weight (MW) cut-off) and snap-frozen in liquid N_2.

MITS9220_PhnD1 protein was screened against multiple crystallisation screens (JCSG+, LMB, SG1, Hampton Index, BCS and BSG) at 20 °C using 96-well in situ plates (Crystal QuickX). Crystals were grown by mixing 100 nl of purified protein with 100 nl of reservoir (0.1 M Tris pH 8.5, 25% PEG 3350) at a concentration of 10 mg/ml. A complete dataset was collected at VMXi from 6 crystals. Data were processed using XDS (Kabsch, 2010) and merged using XSCALE as this data was collected prior to xia2.multiplex being available. The MITS9220_PhnD1 RT structure was solved by MR using the

coordinates of MITS9220_PhnD1 cryo structure. Final refinement statistics are given in Table 2, and coordinates are deposited in the PDB (7ZCK).

S2.3. Bovine AbD08 antibody

Bovine immunoglobulin variable domain forward primers, and IgM, IgG, Igλ and Igκ reverse primers were designed using the Bovine IgH 202 BAC assembly (Ma *et al.*, 2016). Bovine antibody H and L chains were amplified from sorted single B cells by semi-nested PCR using a OneStep RT-PCR Kit (Qiagen) and sequenced by Sanger sequencing (Eurofins). Synthetic genes encoding the variable regions of the naïve ultralong bovine antibody (naïve ultralong VH and VL) were purchased from IDT Technology as gBlocks and inserted into the pOPINBOVH and pOPINBOVL expression vectors respectively (Ren *et al.*, 2019). All vectors were sequenced to confirm clones were correct. Recombinant Fabs were produced by co-transfection of VH and VL vectors into Expi293™ cells according to the manufacturer's protocol (Invitrogen). Proteins were purified from culture supernatants by a combination of immobilised metal affinity and size-exclusion chromatography using an automated protocol implemented on an ÄKTAxpress (GE Healthcare) (Nettleship *et al.*, 2009). Eluted fractions were characterised by SDS-PAGE, and those of interest were spin concentrated using Amicon® Ultracel-30 centrifugal filters (Merck).

Protein crystallizations were carried out using standard OPPF protocols (Walter *et al.*, 2005). Naïve ultralong bovine antibody crystals were grown in a variety of different buffer conditions containing 15% (v/v) PEG 550 MME, 7.5% (v/v) PEG 20000, though the highest diffracting crystals appeared in the presence of 0.09 M Sodium nitrate, 0.09 M Sodium Phosphate dibasic, 0.09 M Ammonium sulphate and 0.1 M Tris (base), 0.1 M BICINE at pH 8.5 by mixing 100 nl of purified protein with 100 nl of reservoir at a concentration of 23 mg/ml. Crystals were screened by their morphology, and absorbance of 280 nm light, and *in-situ* X-Ray diffraction data were collected at 295 K using a Dectris Eiger2 X 4M detector on beamline VMXi at Diamond Light Source (Sanchez-Weatherby *et al.*, 2019). Crystal quality resulting from each well was assessed by these metrics to select crystals for cryogenic data collection. For the room temperature *in-situ* data collection, diffraction was observed at each 0.1° oscillation angle over ±25° total plate oscillation, where each image was exposed for 0.0018 s with 2% beam transmission. A complete dataset was collected to 2.20 Å from combining 4 room-temperature crystal datasets using VMXi and was characterised as belonging to the $P\,2_1\,2_1\,2_1$ space group, with unit cell dimensions of a = 64.22 Å, b = 71.58 Å, c = 100.23 Å, and angles of $\alpha = \beta = \gamma = 90°$. Data merging was performed in AIMLESS (Evans & Murshudov, 2013) and R_{free} was selected randomly for 5% of the reflections. Molecular replacement was performed in PHASER (McCoy *et al.*, 2007), using each chain monomer of the AbD08 cryo-structure as search models. MR placed 1 monomer in the ASU. This phasing solution was used with ARP/wARP (Perrakis *et al.*, 2001) for automated model building which were refined with 50 cycles of rigid body refinement in Refmac (Murshudov *et al.*, 2011) with

resolution range 58.32 - 3 Å, followed by restrained refinement using the entire resolution range. Water molecules were added only at the end of the refinement automatically above 3.0 Fo-Fc in COOT (Emsley & Cowtan, 2004) and manually below 3.0 Fo-Fc. Re-iterated model building was performed in COOT and lead to a final R_{factor} and R_{free} of 17.7% and 22.8%. Model geometry was assessed using MOLPROBITY (Williams *et al.*, 2018).

For the cryogenic data collection, selected crystals were cryocooled in liquid nitrogen and X-Ray diffraction data were collected at 100 K using a Dectris Eiger2 XE 16M detector on beamline I04 at Diamond Light Source. A single, cryo-cooled AbD08 crystal diffracted to 1.59 Å, and was characterised as belonging to the $P\ 2_12_12_1$ space group, with unit cell dimensions of a = 63.50 Å, b = 71.11 Å, c = 98.41 Å, and angles of $\alpha = \beta = \gamma = 90°$ by the xia2 3dii pipeline. Data were collected at each 0.1° oscillation angle over 180° total oscillation, where each image was exposed for 0.008 s with an exposure flux of 4.25 $x10^{11}$ photons. All images were selected for data reduction in XDS (Kabsch, 2010) as implemented in XIA2 (Winter, 2010), as part of the Diamond Light Source automated pipeline. Data merging was performed in AIMLESS (Evans & Murshudov, 2013) and R_{free} was selected randomly for 5% of the reflections. Molecular replacement was performed in PHASER (McCoy *et al.*, 2007), using the protein chain H monomer of bovine ultralong antibody BLV1H12 (PDB : 4K3D) and the protein chain L monomer of bovine antibody B4HC-B13LC (PDB: 6QN7) as searching models. MR placed 1 monomer in the ASU. This phasing solution was used with ARP/wARP (Perrakis *et al.*, 2001) for automated model building which were refined with 50 cycles of rigid body refinement in Refmac (Murshudov *et al.*, 2011) with resolution range 57.64 - 3 Å, followed by restrained refinement using the entire resolution range. Water molecules were added only at the end of the refinement automatically above 3.0 σ and manually below 3.0 Fo-Fc. Re-iterated model building was performed in COOT and lead to a final R_{factor} and R_{free}(Williams *et al.*. Figures were prepared using PyMOL(Yuan *et al.*. Final refinement statistics are given in Table 2 and coordinates deposited in the PDB (8BS8).

S2.4. Human NCOA7

The Δ 1-171bp *ncoa7* gene (Uniprot code for full gene: H0Y5F6) was synthesised and codon optimised for *E. coli* (GeneArt, Invitrogen) to obtain D57aa-NCOA7 protein, as the first 57 aa at the N-terminal were predicted to be disordered based on PONDR analysis (Xue *et al.*, 2010). The D1-171bp ncoa7 gene was sub-cloned into the pET15b vector (Novagen) using the BamHI and NdeI restriction sites. DNA sequencing (Eurofins) confirmed the correct insertion. The DNA was amplified in DH5a *E. coli* strain (Invitrogen) and transformed into *E. coli* BL21 (DE3) (Novagen) expression strain for selection on LB agar supplemented with ampicillin at final concentration of 100 μg/mL. Overnight bacterial

cultures were inoculated into 500 mL of 2YT medium (Melford) in dilution 1:100 v/v at 37°C with the same antibiotic and protein production was induced when OD_{660nm} reached 0.7 by addition of IPTG at a final concentration of 0.005 M (Generon). Bacterial growth was performed at 18°C for 18 hrs and bacteria were harvested by centrifugation at 7,000 g for 10 min. Cell pellet was resuspended in buffer A (0.05 M NaPi, 0.5 M NaCl, pH 8) supplemented with 0.03 M imidazole and one EDTA-free protease inhibitor tablet (Roche). Cell lysis was carried out by sonication and the lysate spun down at 50,000 x g for 30 min. Supernatant fraction was loaded into a Co^{2+}-NTA resin (Thermo -Fisher) and protein eluted in buffer A supplemented with 0.5 M imidazole. The protein-containing fractions were pooled together and dialysed overnight against buffer B (0.02 M HEPES, 0.15 M NaCl, pH 8). The protein sample was concentrated using Millipore concentrators (10 kDa MW cut-off) and injected into a S75 16/600 column (Cytiva) equilibrated with buffer B supplemented with 0.005 M TCEP. Protein-containing fractions were analysed on SDS-PAGE and pooled together to be concentrated to 7.9 mg/mL using Millipore concentrators (10 kDa MW cut-off). Δ57-NCOA7 protein sample was flash-cooled in liquid N_2 and shipped on dry ice to VMXi for screening.

Δ57-NCOA7 protein sample was screened against multiple crystallisation screenings (JCSG+, LMB, SG1, Hampton Index, BCS and BSG) at 18°C using 96-well in situ plates (Mitegen) by mixing 50 nL of protein with 100 nL of reservoir solution. The protein crystallised in 3 days at 18°C in several conditions: 0.05 M Zinc acetate dihydrate, 20% w/v PEG 3350 (condition 1); 0.2 M di ammonium hydrogen citrate, 20% w/v PEG 3350 (condition 2); 0.1 M citrate pH 5, 20% w/v PEG 6000 (condition 3); 0.2 M NH_4S0_4, 0.1 M Na acetate pH 4.6 and 25% w/v PEG 4000 (condition 4); 1.5 M Lithium sulfate, 0.1 M Sodium HEPES pH 7.5 (condition 5); 0.2 M Sodium sulfate, 20% w/v PEG 3350 (condition 6), 8% w/v, PEG 8000, 0.08 M Potassium phosphate pH 5.6 (condition 7). The best diffracting crystals were in bi-pyramidal (crystal form I) or plate morphology (crystal form II) (Figure 2C and 2D) and obtained in condition 2, often present in the same crystallisation drop.

Crystal form I belonged to space group (SG) $P4_12_12$ with unit cell parameters (a = 89.74 Å, b= 89.74 Å, c= 166.19 Å, α =β=γ= 90°) and diffracted to 3.0 Å at RT and to 2.2 Å, whilst crystal form 2 belonged to SG C2 with cell unit cell parameters (a = 100.0 Å, b = 54.8 Å, c= 44.2 Å, α = 90°, β = 119.5°, γ = 90°) and diffracted to 2.36 Å at RT . A complete dataset was collected from crystal form I to 2.36 Å from 12 crystals using the automated pipeline at VMXi. Molecular replacement was performed in PHASER (McCoy *et al.*, 2007), using the protein monomer of zebrafish homologue as a search model (PDB: 4ACJ) (Blaise *et al.*, 2012) after side chain pruning in Sculptor as implemented in PHENIX (Adams *et al.*, 2011). MR placed three monomers in the ASU. which were refined with 10 cycles of rigid body in Refmac (Murshudov *et al.*, 2011) with resolution range 10-4 Å, followed by restrained refinement using the entire resolution range. Water molecules were added only at the end of the refinement automatically above 3.0 r.m.s.d Fo-Fc in COOT (Emsley & Cowtan, 2004) and manually below 3.0 r.m.s.d Fo-Fc. Iterative model building was performed in COOT and lead to a final R_{factor} and

R_{free} of 19.09% and 23.79% for the cryo structure in $P4_12_12$. Same data reduction, molecular replacement and refinement strategies were followed with the RT dataset of crystal form II leading to R_{factor} and R_{free} of 19.6% and 25.1% for the RT structure in C2. Model geometry was assessed using MOLPROBITY (Williams *et al.*, 2018). Figures were prepared using PyMOL (Yuan *et al.*, 2016).

S2.5. Cytochromes c': PHCP and TTCP

The gene for the mature PHCP protein fused with the gene for the *Rhodopseudomonas palustris* cytochrome c_{556} signal peptide at the N-terminus flanked by *Eco*RI and *Sal*I restriction sites was synthesized with codon optimization for expression in *E. coli* by a manufacturer (Operon, Japan). The resulting gene was inserted into the EcoRI and SalI sites of the pKK223-3 vector under the control of the *tac* promoter. The resulting plasmid was transformed into *E. coli* strain JCB387 carrying the pEC86 plasmid encoding cytochrome *c* maturation proteins. The transformed *E. coli* cells were grown aerobically and a periplasmic protein fraction containing the recombinant PHCP protein was extracted by the cold osmotic shock method (Sambongi *et al.*, 1996). The periplasmic fraction was dialyzed against 0.010 M Tris-HCl buffer (pH 8 at 4 °C), and then loaded onto a Hi-Trap Q column (diameter, 1.4 cm; height, 2.5 cm) that had equilibrated with the same buffer. The protein was eluted with a linear gradient of NaCl (0-0.2 M). The brown fraction was dialyzed against 0.025 M sodium acetate buffer pH 5.5, and then loaded onto a Hi-Trap SP column (diameter, 1.4 cm; height, 2.5 cm) that had been equilibrated with the same buffer. The PHCP protein was flowed through the column. The protein fraction was dialyzed against 0.01 M Tris/HCl pH 8.0 containing 0.15 M NaCl, and them finally loaded onto Superdex 75 column (diameter, 1.6 cm; height, 60 cm). The protein purity was checked by SDS-PAGE.

The gene for the mature TTCP protein fused with the gene for the *Pseudomonas aeruginosa* cytochrome c_{551} signal peptide at the N-terminus flanked by *Eco*RI and *Sal*I restriction sites was synthesized with codon optimization for expression in *E. coli* by a manufacturer (ThermoFisher Scientific, US). The resulting gene was inserted into the EcoRI and SalI sites of the pKK223-3 vector under the control of a *tac* promoter. The resulting plasmid was transformed into *E. coli* strain JCB387 carrying the pEC86 plasmid encoding cytochrome *c* maturation proteins. The transformed *E. coli* cells were grown aerobically and a periplasmic protein fraction containing the recombinant TTCP protein was extracted by the cold osmotic shock method (Sambongi *et al.*, 1996). The periplasmic protein fraction containing the TTCP protein was dialyzed against 0.01 M Tris-HCl buffer (pH 8 at 4°C), and then loaded onto a HiTrap Q anion-exchange column (diameter, 1.4 cm; height, 3 cm). The TTCP protein was eluted with the same buffer containing a linear gradient of NaCl concentrations from 0 to 0.3 M. The fractions containing the TTCP protein were then dialyzed against 0.025 M sodium acetate buffer (pH 5 at 4°C) and loaded onto a HiTrap SP cation-exchange column (diameter, 1.4 cm; height,

2.5 cm). The TTCP protein was eluted with the same buffer containing a linear gradient of NaCl concentrations from 0 to 0.3 M. The fractions containing the TTCP protein were dialyzed against 0.01 mM Tris-HCl buffer (pH 8 at 4°C) plus 0.15 M NaCl, and loaded onto a Superdex 75 column (diameter, 1.6 cm; height, 60 cm), which was equilibrated and then eluted with the same solution. The protein purity was checked by SDS-PAGE.

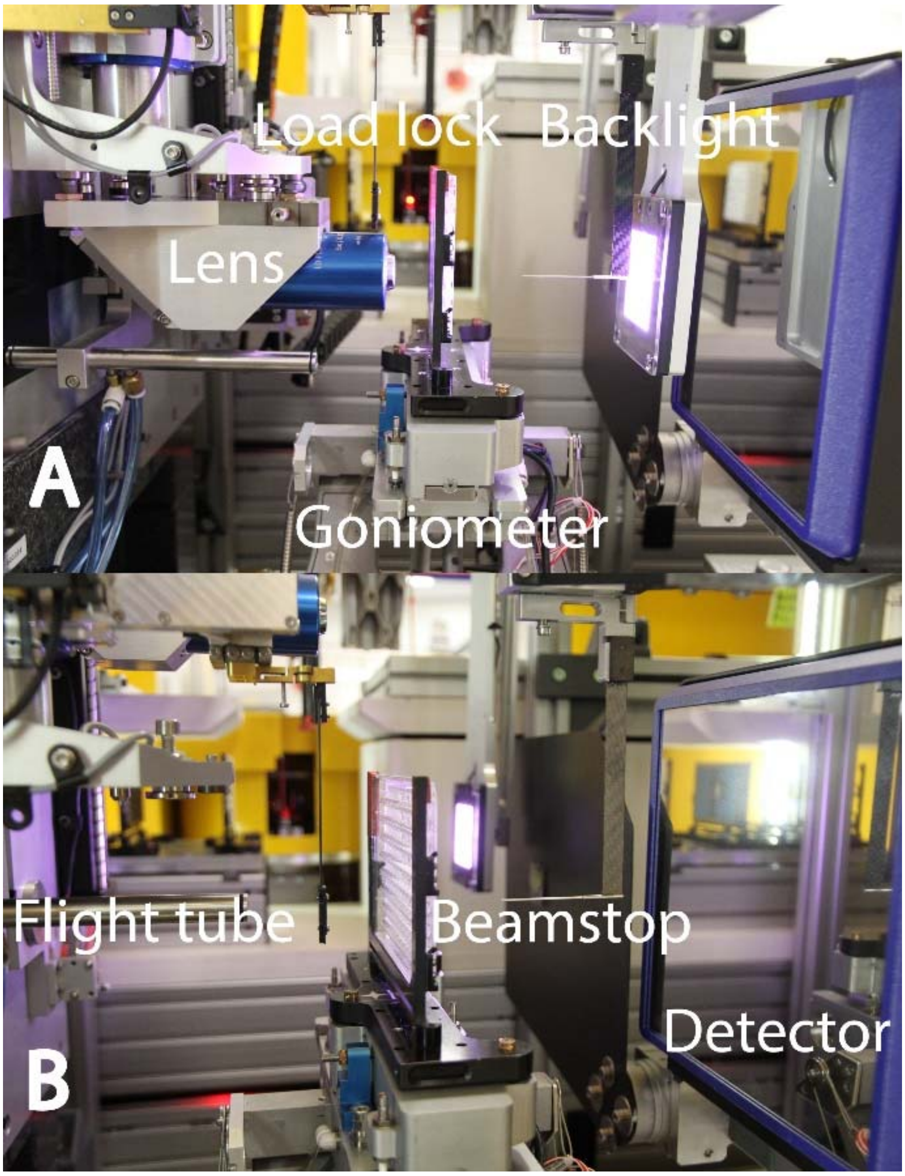

Figure S1 The VMXi endstation showing a plate mounted on the goniometer in (A) imaging, and (B) data collection modes. Crystallisation plates are delivered from a Formulatrix Rock Imager unit to the beamline using a Mitsubishi robotic arm via a load lock. A second robotic arm inserts plates into holders within local storage, which also facilitates movement between local storage and high-precision goniometer. A high-definition camera system takes images of each crystallisation drop and an image-matching algorithm allows motor positions to be generated which correlate with the points or regions of interest marked by users. This allows the accurate sample centring of objects ~10 μm and larger, with the 10 x 10 μm X-ray beam at sample position. Oscillation data are typically collected in a 60° sweep using a Dectris Eiger2 X 4M detector. A high-resolution lens for producing the sample images can be seen in panel A, which is replaced by a helium-filled flight tube (to reduce air scatter) shown in panel B, for data collection.

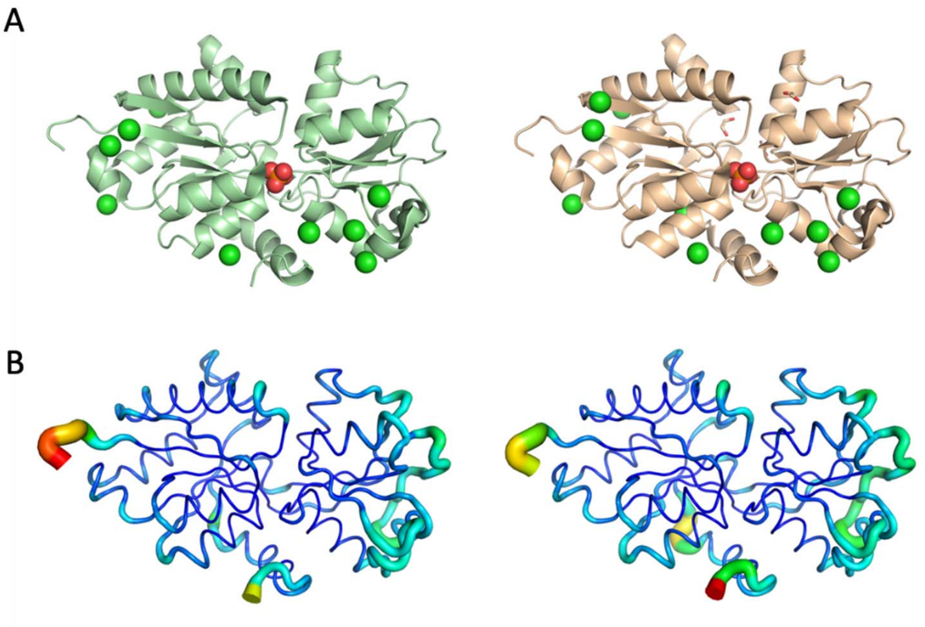

Figure S2 Comparison of MITS9220_PhnD1 RT and 100 K structures **(A)** Cartoon representation of MITS9220_PhnD1 RT (7ZCK; green) and 100 K (7S6G; orange) structures. Phosphate (red spheres) and Cl- ions (green spheres) sequestered from the crystallisation condition are depicted. **(B)** B-factor putty representation of MITS9220_PhnD1 RT (left) and 100 K (right) structures.
